# Overexpression of Toll-like receptor 8 correlates with the progression of podocyte injury in murine autoimmune glomerulonephritis

**DOI:** 10.1038/srep07290

**Published:** 2014-12-03

**Authors:** Junpei Kimura, Osamu Ichii, Kosuke Miyazono, Teppei Nakamura, Taro Horino, Saori Otsuka-Kanazawa, Yasuhiro Kon

**Affiliations:** 1 Laboratory of Anatomy, Department of Biomedical Sciences, Graduate School of Veterinary Medicine, Hokkaido University, Sapporo, Japan; 2 Laboratory of Molecular Medicine, Department of Veterinary Clinical Sciences, Graduate School of Veterinary Medicine, Hokkaido University, Sapporo, Japan; 3 Section of Biological Safety Research, Chitose Laboratory, Japan Food Research Laboratories, Chitose, Japan; 4 Department of Endocrinology, Metabolism and Nephrology, Kochi Medical School, Kochi University, Nankoku, Japan

## Abstract

Members of the Toll-like receptor (TLR) family serve as pathogen sensors and participate in local autoimmune responses. This study found a correlation between glomerular injury and TLR expression by analysing BXSB/MpJ-*Yaa* (BXSB-*Yaa*) lupus model mice. In isolated glomeruli, the mRNA expression of several TLRs was higher in BXSB-*Yaa* mice than in healthy control BXSB mice. In particular, the expression of *Tlr8* and its downstream cytokines was markedly increased. In mouse kidneys, TLR8 protein and mRNA localized to podocytes, and TLR8 protein expression in the glomerulus was higher in BXSB-*Yaa* mice than in BXSB mice. In BXSB-*Yaa* mice, the glomerular levels of *Tlr8* mRNA negatively correlated with the glomerular levels of podocyte functional markers (*Nphs1, Nphs2,* and *Synpo*) and positively correlated with urinary albumin levels. Furthermore, the glomerular and serum levels of miR-21, a putative microRNA ligand of TLR8, were higher in BXSB-*Yaa* mice than in BXSB mice. The urinary levels of *Tlr8* mRNA were also higher in BXSB-*Yaa* mice than in BXSB mice. In conclusion, the overexpression of TLR8 correlates with the progression of podocyte injury in glomerulonephritis. Thus, altered levels of urinary *Tlr8* mRNA might reflect podocyte injury.

Systemic lupus erythematosus (SLE) is an autoimmune disease characterized by autoantibody production and immune complex deposition that result in tissue inflammation and damage^1^. SLE-related glomerulonephritis (GN), also known as lupus nephritis (LN), is one of the most common and severe complications of SLE because of the risk of cardiovascular disease and end-stage renal disease^2^.

NZB, (NZB × NZW) F1 hybrid, BXSB/MpJ-*Yaa* (BXSB-*Yaa*), and MRL/MpJ-*lpr* mice are commonly used as spontaneous SLE models^3^. These strains develop systemic autoimmune diseases characterized by increased serum autoantibody levels and vasculitis, in addition to GN that is similar to human LN^3^. Recently, we described the pathological interactions between the immune-associated genes on chromosome 1 and the genetic locus on chromosome Y in the glomerular pathogenesis of BXSB-*Yaa* mice^4^*.* BXSB-*Yaa* mice carry a genetic mutation located on the Y chromosome, namely, Y-linked autoimmune acceleration (*Yaa*). The severity of GN is greater in males than in females because of the *Yaa* mutation^3^,^4^,^5^,^6^. The *Yaa* mutation is a translocation from the telomeric end of the X chromosome to the Y chromosome. The duplicated segment plays a crucial role in the activation of auto-reactive B cells, thereby contributing to the *Yaa*-mediated enhancement of the autoimmune phenotype in male BXSB-*Yaa* mice^7^. The *Yaa* locus contains several immune-associated genes, including Toll-like receptor (TLR) family members^7^.

TLRs are expressed on the plasma membrane or intracellular vesicular membrane of hematopoietic and non-hematopoietic cells^8^. They have been characterized as innate immune sensors that recognize danger signals arising from pathogen-associated molecular patterns (PAMPs), including flagellin, lipopolysaccharide (LPS), and nucleic acids derived from bacteria, mycobacteria, mycoplasma, fungi, and viruses^8^. Previous studies have identified 12 members of the TLR family in mice (TLR1–9 and TLR11–13) and 10 in humans (TLR1–10)^8^. When activated by their own pathogenic ligands, TLRs enhance inflammatory cytokine expression mainly through the NF-κΒ pathway to provide host defence^8^.

Interactions between TLRs and their endogenous ligands have been shown to play important roles in the pathogenesis of non-infectious injury^9^,^10^,^11^,^12^. Mersmann *et al.* have suggested that endogenous high-mobility group box 1 (HMGB1) contributes to myocardial injury through the activation of TLR2 signalling^11^. Shichita *et al*. have demonstrated a pathological interaction between endogenous peroxiredoxin and TLR2 or TLR4 on macrophages in ischemic brain injury^12^. Endogenous TLR ligands are called danger-associated molecular patterns (DAMPs) and are thought to be danger signals that relay the presence of tissue injury to immune cells or local intrinsic cells, thereby inducing local tissue inflammation and damage^9^,^10^,^11^,^12^.

Experimental and clinical studies have shown that TLRs expressed in intrinsic renal cells are involved in the pathogenesis of several kidney diseases^13^,^14^,^15^,^16^,^17^,^18^. In particular, TLR4, TLR5, and TLR11 in tubular epithelial cells play an important role in the pathogenesis of urinary tract infections and sepsis-induced renal failure^14^,^15^,^16^. The activation of TLR2 or TLR4 by DAMPs in tubular epithelial cells contributes to the progression of kidney ischemia-reperfusion injury and subsequent renal fibrosis^17^,^18^. This suggests that activation of the TLR signalling pathway plays a crucial role in renal tubulointerstitial injury in various pathological conditions. However, little is known about the involvement of TLRs in glomerular diseases. In the present study, we focused on LN and found marked upregulation of *Tlr8* and its downstream cytokines in the glomeruli of BXSB-*Yaa* mice.

## Results

### Clinical parameters of BXSB-*Yaa* mice

With regard to the clinical index of the systemic autoimmune condition, serum anti-double-strand DNA (dsDNA) antibody levels were higher in BXSB-*Yaa* mice than in BXSB/MpJ-*Yaa*^+^ (BXSB) mice at 2 and 4 months of age (Table 1). There were no differences in the indices of renal function, including serum blood urea nitrogen (sBUN) and serum creatinine (sCre), between the strains at any age. However, urinary albumin-to-creatinine ratio (uACR) levels, which serve as an index of glomerular dysfunction, were higher in BXSB-*Yaa* mice than in BXSB mice at 4 months of age (Table 1).

### Glomerular histopathology in BXSB-*Yaa* mice

**Figure 1:**
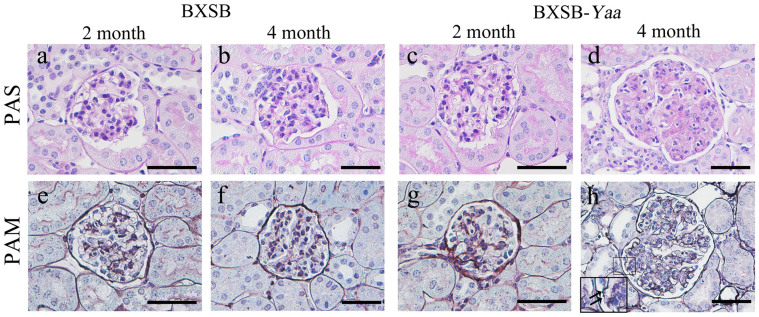
Glomerular histopathology of BXSB-*Yaa* mice. (a–d) Histopathology of glomeruli in periodic acid-Schiff (PAS)-stained sections from BXSB and BXSB-*Yaa* mice. In BXSB mice (a and b), there are no histological differences at the ages of 2 and 4 months. In BXSB-*Yaa* mice (c and d), mesangial matrix expansion and mesangial cell proliferation are clearly observed at 4 months, but not at 2 months. (e–h) Histopathology of glomeruli in periodic acid methenamine silver (PAM)-stained sections from BXSB and BXSB-*Yaa* mice. In BXSB mice (e and f), there are no histological differences at 2 and 4 months. In BXSB-*Yaa* mice (g and h), glomerular hypertrophy, wrinkling of the glomerular basement membrane (GBM), and spike-like structures of the GBM (inset, arrows) are clearly observed at 4 months (h). Bars = 50 μm.

Glomerular histopathology was examined in kidney sections stained with periodic acid-Schiff (PAS) (Fig. 1a–d) or periodic acid methenamine silver (PAM) (Fig. 1e–h) at 2 and 4 months of age. No glomerular lesions were observed at any age in BXSB mice (Fig. 1 a, b, e, and f) or at 2 months in BXSB-*Yaa* mice (Fig. 1c and g). In contrast, at 4 months of age, BXSB-*Yaa* mice developed GN, which was characterized by glomerular hypertrophy, increases in mesangial cell number and the mesangial matrix, thickening of the glomerular basement membrane (GBM), and spike-like structures on the GBM (Fig. 1d and h).

### Glomerular expression of TLR family members and activation of TLR-mediated signalling in BXSB-*Yaa* mice

**Figure 2:**
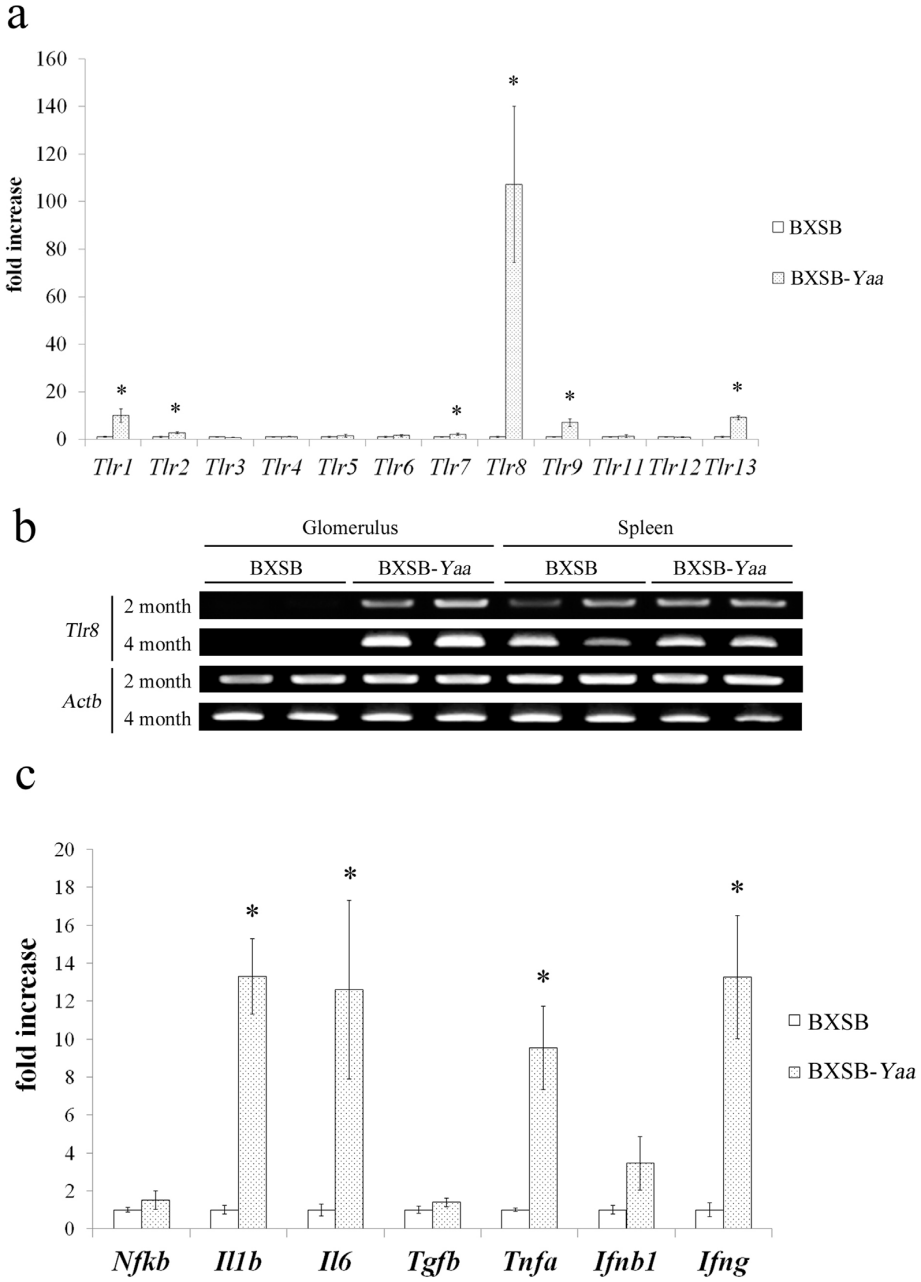
mRNA expression of TLR family members and their downstream factors in the glomeruli of BXSB-*Yaa* mice. (a) Relative mRNA expression of TLR family genes in isolated glomeruli from BXSB-*Yaa* and BXSB mice at 4 months. The expression levels were normalized to the levels of *Actb*. Values are the mean ± s.e. *, significantly different from control BXSB mice (Mann-Whitney *U*-test, *P* < 0.05); n ≥ 4. (b) RT-PCR analysis of *Tlr8* and *Actb* mRNA expression in the glomeruli and spleen of BXSB and BXSB-*Yaa* mice at 4 months of age. n = 2. (c) Relative mRNA expression of *Nfkb*, *Il1b*, *Il6*, *Tgfb*, *Tnfa*, and *Ifnb1* in isolated glomeruli from BXSB-*Yaa* and BXSB mice at 4 months of age. The expression levels were normalized to the levels of *Actb*. The values are the mean ± s.e. *, significantly different from control BXSB-*Yaa* mice (Mann-Whitney *U*-test, *P* < 0.05); n ≥ 4.

To determine which TLR members are associated with GN pathogenesis, we first examined the expression of 12 TLR family genes in the isolated glomeruli of BXSB-*Yaa* mice at 4 months of age (Fig. 2a). The glomerular expression of *Tlr1*, *2*, *7*, *8*, *9*, and *13* was higher in BXSB-*Yaa* mice than in BXSB mice. In particular, *Tlr8* expression increased markedly (108-fold, *P* < 0.001). Semi-quantitative RT-PCR analysis (Fig. 2b) showed that glomerular *Tlr8* expression was higher in BXSB-*Yaa* mice than in BXSB mice at 2 and 4 months of age and that the *Tlr8* band intensity was stronger at 4 months than at 2 months in the glomeruli of BXSB-*Yaa* mice. An increase in glomerular *Tlr8* expression was also observed in B6.MRLc1(68-81) mice, which comprise an autoimmune GN model that we established previously^19^ (Supplementary Fig. 1). Because of these findings, we focused on TLR8 in subsequent analyses.

Figure 2c shows the glomerular expression of inflammatory mediators induced by the activation of TLRs^13^. The glomerular expression of inflammatory cytokines in the NF-κB pathway, including interleukin 1 beta (*Il1b*)*, Il6*, and tumour necrosis factor (*Tnfa*), was higher in BXSB-*Yaa* mice than in BXSB mice at 4 months of age. In contrast, there were no differences between the strains in the expression of *Nfkb*, transforming growth factor beta (*Tgfb*), and interferon beta 1 (*Ifnb1*).

### Localization of TLR8 in mouse and human kidneys

**Figure 3:**
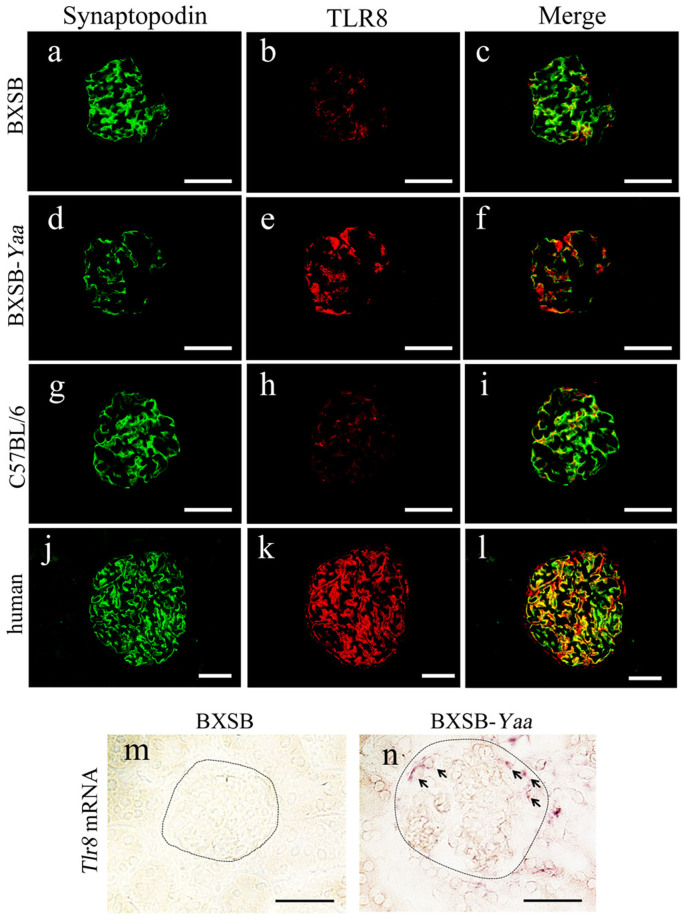
Localization of TLR8 protein and mRNA in the kidneys of mice and humans. (a–l) Immunofluorescence of synaptopodin (a, d, g, and j) and TLR8 (b, e, h, and k) in the glomeruli of BXSB (a–c), BXSB-*Yaa* (d–f), and C57BL/6 (g–i) mice and humans (j–l). Merged images are shown (c), (f), (i), and (l). Synaptopodin immunoreactivity (green) co-localized with that of TLR8 (red) (c, f, i, and l). TLR8 positivity in BXSB-*Yaa* mice (e) is stronger than that in BXSB (b) and C57BL/6 (h) mice*.* In the human glomerulus (j–l), TLR8 immunoreactivity co-localizes with synaptopodin immunoreactivity, as in the mouse glomerulus. (m and n) *In situ* hybridization for *Tlr8* mRNA. Positive reactions are observed in the glomeruli of BXSB-*Yaa* cells, especially in podocyte regions (n, arrow). In contrast, no positive reaction is observed in the BXSB glomerulus (m). Bars = 50 μm.

In immunofluorescence analysis, synaptopodin, a podocyte marker, was detected in the podocyte regions of all examined mice (Figs. 3a, d, and g). However, synaptopodin immunoreactivity was weaker in BXSB-*Yaa* mice (Fig. 3d) than in BXSB and C57BL/6 mice (Fig. 3a and g) at 4 months of age. TLR8 was observed along the glomerular capillary rete, especially in podocyte regions (Fig. 3b, e, and h). The immunoreactivity was stronger in BXSB-*Yaa* mice (Fig. 3e) than in the other two strains (Fig. 3b and h) at 4 months of age. TLR8 co-localized with synaptopodin in the glomeruli of all examined strains (Fig. 3c, f, and i). Although synaptopodin positivity was lower in BXSB-*Yaa* mice (Fig. 3d), we still detected co-localization of synaptopodin with TLR8 (Fig. 3f). As observed in mice, TLR8 co-localized with synaptopodin in healthy human kidneys (Fig. 3j, k, and l).

In *in situ* hybridization analysis of *Tlr8* mRNA, signal was not detected in the glomeruli of BXSB mice (Fig. 3m). In contrast, signal localized to the podocyte region in the glomeruli of BXSB-*Yaa* mice (Fig. 3n).

### Correlation between podocyte injury and *Tlr8* mRNA expression in BXSB-*Yaa* mice

The correlations between indices of podocyte injury and *Tlr8* mRNA expression in isolated glomeruli were analysed in BXSB-*Yaa* mice at 4 months of age (Table 2). Glomerular *Tlr8* mRNA levels positively correlated with uACR levels, a functional index of glomerular injury, in BXSB-*Yaa* mice (Spearman's test, *P* < 0.01). Furthermore, glomerular *Tlr8* mRNA levels negatively correlated with the glomerular mRNA levels of podocyte functional markers, including nephrin (*Nphs1*), podocin (*Nphs2*), and synaptopodin (*Synpo*), in BXSB-*Yaa* mice.

### Glomerular and serum levels of a putative endogenous ligand of TLR8 in BXSB-*Yaa* mice

**Figure 4:**
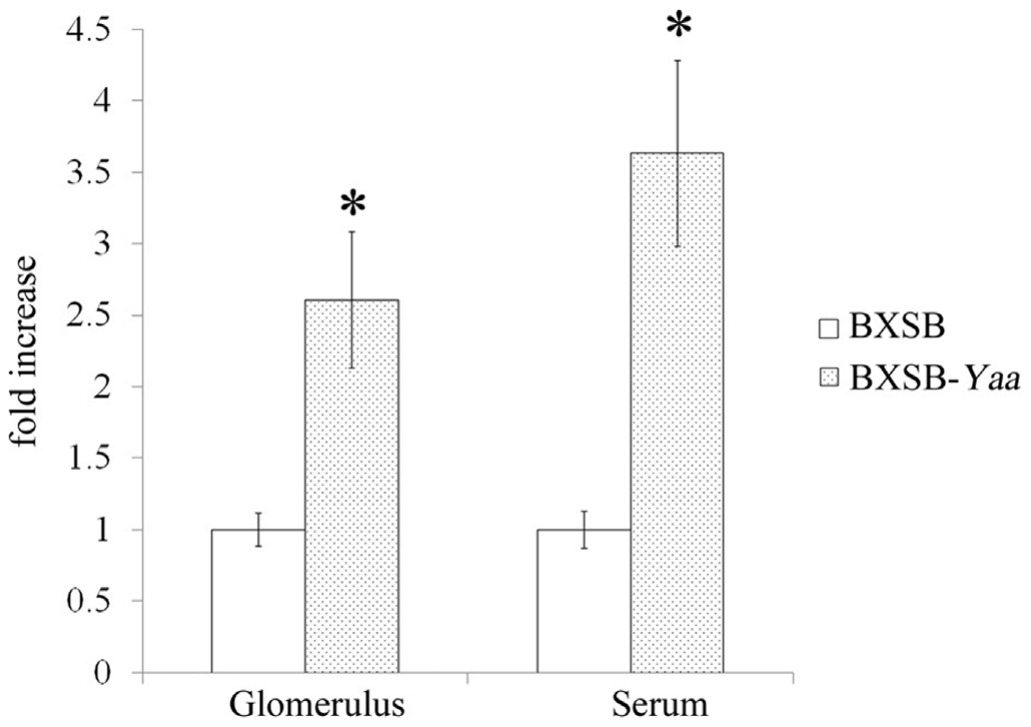
Glomerular and serum levels of miR-21. The relative glomerular and serum expression of miR-21 in BXSB and BXSB-*Yaa* mice at 4 months of age. Values are the mean ± s.e. Data are presented as the fold increase vs. BXSB in the same samples. *, significantly different from control BXSB mice (Mann-Whitney *U*-test, *P* < 0.05); n = 3.

A recent study reported that microRNAs, particularly miR-21, act as ligands for TLR8^20^. We next examined the glomerular and serum levels of miR-21. miR-21 levels were higher in BXSB-*Yaa* mice than in BXSB mice at 4 months of age (Fig. 4).

### Detection of *Tlr8* mRNA in the urine of BXSB-*Yaa* mice

**Figure 5:**
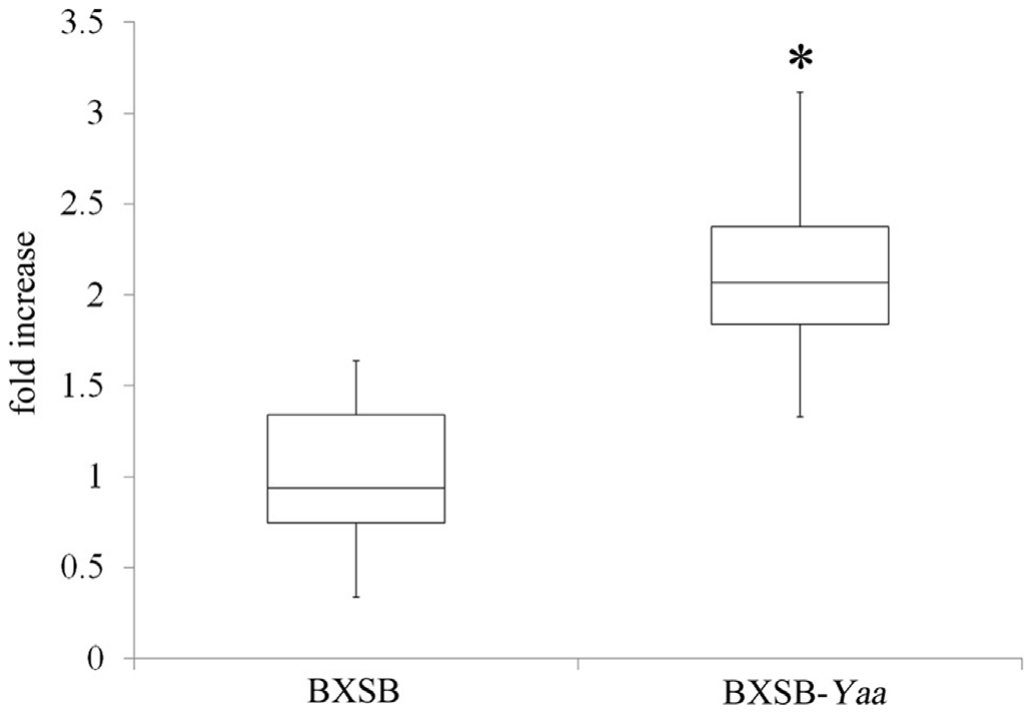
Urinary levels of *Tlr8* mRNA. Box plots of the relative *Tlr8* mRNA levels in urine from BXSB and BXSB-*Yaa* mice. Values are the mean ± s.e. Data are presented as the fold increase vs. BXSB. *, significantly different from control BXSB mice (Welch's t-test, *P* < 0.05); n ≥ 4.

The urinary *Tlr8* mRNA levels of BXSB-*Yaa* mice and control mice were determined (Fig. 5). The urinary *Tlr8* levels were higher in BXSB-*Yaa* mice than in BXSB mice at 4 months of age. On the other hand, we detected no difference between serum *Tlr8* levels in BXSB-*Yaa* mice and BXSB mice (Supplementary Fig. 2).

## Discussion

Among glomerular cells, TLRs are expressed by mesangial cells (TLR2–4), endothelial cells (TLR2, 4, 9), and podocytes (TLR1–6, 8, 9)^13^,^21^. Pawar *et al*. have demonstrated that the administration of poly (I:C), the ligand for TLR3, aggravates autoimmune GN in MRL/MpJ-*lpr* mice, whereas this ligand does not alter anti-DNA autoantibody levels and does not induce B cell activation^22^. According to Fu *et al*., anti-GBM antibody-treated mice develop mild GN, but when the treatment is coupled with specific TLR ligands, including peptidoglycan (TLR2), poly (I:C) (TLR3), LPS (TLR4), or flagellin (TLR5), the treated mice developed GN of greater severity associated with the activation of the NF-κΒ pathway^23^. Thus, several *in vivo* studies suggest that TLRs and their exogenous ligands have pathogenic roles in GN^13^,^22^,^23^.

In the present study, we demonstrated that TLRs, including *Tlr1*, *2*, *7*, *8*, *9,* and *13*, and their downstream factors (*Il1b*, *Il6,* and *Tnfa*) were upregulated through the NF-κΒ pathway in the glomeruli of autoimmune GN mice. From these findings, we concluded that the TLR-mediated NF-κΒ pathway plays an important role in the pathogenesis of autoimmune GN. Importantly, the mRNA expression of TLR family members was induced by major cytokines such as interferon gamma (IFNγ) and TNFα in both inflammatory cells and tissue-intrinsic cells^17^,^24^. Experimental and clinical studies have shown that IFNγ and TNFα are upregulated in the serum and kidneys of SLE patients and SLE-prone mice^25^,^26^. Indeed, we detected higher levels of glomerular *Tnfa* and *Ifng* in BXSB-*Yaa* mice than in control mice (Fig. 2c). Collectively, these results indicate that local cytokines increased local *Tlr8* expression, especially in podocytes. A previous study showed that *Tlr8* is broadly expressed on myeloid dendritic cells, monocytes, differentiated macrophages, and CD4^+^ regulatory T cells^27^. Podocytes might have differential sensitivity to *Tlr8*-inducing cytokines when compared to immunocompetent cells, and this might contribute to the overexpression of glomerular *Tlr8*. Thereby, *Tlr8* overexpression in podocytes would enhance the cells' responsiveness to their own ligands. These processes might aggravate the pathological conditions of autoimmune GN.

In a previous study, we found that the BXSB-type genome causes SLE-like symptoms and subsequent GN without involvement of *Yaa* and that *Yaa* accelerates disease progression^4^. Recently, the *Yaa* mutation was characterized as a translocation from the telomeric end of the X chromosome to the Y chromosome^7^. The duplicated segment contains at least 19 genes, including *Tlr7* and *Tlr8*^7^. We observed local overexpression of *Tlr7* and *Tlr8* in BXSB-*Yaa* glomeruli; expression of *Tlr8* was markedly increased. Importantly, TLR8 mRNA and protein localized to podocytes in BXSB-*Yaa* mice. Furthermore, the B6.MRLc1(68-81) lupus-prone strain also showed increased glomerular *Tlr8* expression with age. These results suggest that the *Yaa* mutation in addition to the autoimmunity-prone genetic background caused glomerular TLR8 overexpression in BXSB-*Yaa* mice. Although Gurkan *et al*. have reported that *Tlr8* mRNA is expressed in a mouse immortalized podocyte cell line^21^, our present study demonstrates for the first time that TLR8 protein is expressed by podocytes *in vivo*.

In contrast to TLR8, TLR7 is mainly expressed on infiltrating inflammatory cells, not on renal intrinsic cells, under physiological and pathological conditions^13^. In addition to TLR8, podocytes reportedly express several other members of the TLR family, as described above, and recent studies have indicated a pathological correlation between the TLR-mediated NF-κB pathway in podocytes and podocyte injury *in vitro*^28^,^29^. Banas *et al*. have shown that TLR4 in podocytes interacts with the innate immune system to mediate glomerular injury^28^. Moreover, Machida *et al*. have suggested that TLR9 expression in podocytes is associated with glomerular disease *in vivo*^29^. Because of their unique localization in the glomerulus, podocytes are continuously exposed to various plasma solutes containing TLR ligands such as PAMPs and DAMPs. Therefore, podocytes might contribute to renal immunosurveillance by the TLR-mediated immune system.

TLR8 localizes in the endosomal membrane and recognizes single-stranded RNA and short, double-stranded RNA from microbial organisms, leading to the production of a variety of NF-κB-mediated cytokines^8^. Several studies have shown that TLR8 also recognizes endogenous miRNAs^8^. Although previous studies have demonstrated that secreted miRNAs, which are generally secreted within exosomes, can regulate gene expression in recipient cells via canonical binding to their target mRNAs^30^, Fabbri *et al*. have shown that exosomal miR-21 and miR-29a can function as ligands for TLR8^20^. SLE patients show elevated serum levels of miRNAs, including miR-21^1^,^31^. Furthermore, Pan *et al*. have shown that miR-21 is overexpressed in CD4^+^ T cells from patients with lupus and MRL/MpJ-*lpr* mice^32^. In the present study, we demonstrated that serum and glomerular miR-21 is overexpressed in autoimmune GN models. These findings indicate that an NF-κB-mediated pathway initiated by the interaction between TLR8 and endogenous ligands, including miR-21, correlates with the pathogenesis of autoimmune GN.

We also found that the glomerular expression of *Tlr8* correlated with the expression of podocyte functional markers and with uACR. These results suggest that the TLR8-mediated pathway closely correlates with podocyte injury. IL-1β, one of the most important cytokines in the NF-κB pathway, is involved in kidney injury; in the glomerulus, IL-1β is mainly produced by podocytes^33^,^34^. Furthermore, recent studies have shown that various inflammatory factors, including IL-1β, induce podocyte injury by reducing the production of podocyte functional markers, especially nephrin^35^,^36^. We found a strong correlation between the glomerular expression of TLR8-mediated cytokines, including *Il1b*, and the expression of podocyte functional markers (Supplementary Table 1). Furthermore, our previous study showed that T cells and B cells infiltrate the BXSB-*Yaa* glomerulus as the disease progresses^6^. These findings indicate that factors downstream of TLR8, such as IL-1β, directly contribute to podocyte injury and promote the glomerular recruitment of leukocytes in autoimmune GN pathogenesis.

The urinary expression of *Tlr8* mRNA was higher in BXSB-*Yaa* mice than in control mice. A recent study has suggested that glomerular injury in proteinuric renal diseases is strongly associated with the effacement of podocytes caused by disruption of foot processes and/or the slit diaphragm; podocyte mRNA is detected in the urine of patients with renal disease^37^. We observed TLR8 expression in human and murine podocytes. Therefore, altered urinary levels of *Tlr8* mRNA might indicate podocyte injury in autoimmune GN.

In conclusion, we showed that members of the TLR family and the TLR8-mediated pathway in particular correlate with podocyte injury in murine autoimmune GN, suggesting that TLR8 is a novel therapeutic and diagnostic target for mouse and human glomerular diseases.

## Methods

### Ethics statement

All animal experiments was approved by the Institutional Animal Care and Use Committee, which convenes at the Graduate School of Veterinary Medicine, Hokkaido University (approval No. 13-0032). The investigators adhered to the Guide for the Care and Use of Laboratory Animals of Hokkaido University, Graduate School of Veterinary Medicine (approved by the Association for the Assessment and Accreditation of Laboratory Animal Care International).

### Animals

Male BXSB-*Yaa* mice and BXSB mice, which carry the C57BL/6-type Y chromosome on a BXSB-*Yaa* background, were purchased from Japan SLC Inc. (Shizuoka, Japan) and assigned to an autoimmune GN model group or a healthy control group at 2–4 months of age. All mice were maintained under specific pathogen-free conditions. The animals were anesthetized (60 mg/kg pentobarbital sodium, administered intraperitoneally), and urine was collected by bladder puncture. After urine collection, the mice were euthanized by exsanguination from the carotid artery, and the serum, kidneys and spleen were collected.

### Sample preparation

The kidneys were fixed in 4% paraformaldehyde (PFA) in 0.1 M phosphate buffer (PB; pH 7.4) at 4°C for histopathological analysis. PFA-fixed paraffin sections (2-μm-thick) were then prepared and used for PAS staining, PAM staining, or immunofluorescence. For *in situ* hybridization, a portion of the kidneys was embedded in Tissue-Tek OCT Compound (Sakura Finetechnical, Tokyo, Japan). The splenic tissue was stored in RNAlater solution (Life Technologies, Carlsbad, CA, USA) for total RNA isolation.

### Human samples

Normal kidney tissues from autopsied humans without renal disease were obtained from KAC Inc. (Tokyo, Japan). The kidney samples were used for immunofluorescence analysis of TLR8.

### Glomerular isolation

Murine glomeruli were isolated as previously described^6^. Briefly, 40 mL of Hank's balanced salt solution (HBSS) containing 8 × 10^7^ Dynabeads (Life Technologies) was perfused from the left ventricle. The kidneys were removed and digested with collagenase A (1 mg/mL; Roche, Basel, Switzerland) and deoxyribonuclease I (100 U/mL; Life Technologies) in HBSS at 37°C for 30 min. The digested tissue was gently pressed through a 100-μm cell strainer (BD Falcon, Franklin Lakes, NJ, USA) using a flattened pestle, and the cell suspension was centrifuged at 200 × *g* for 5 min. The cell pellet was resuspended in 2 mL of HBSS. Finally, glomeruli containing Dynabeads were collected using a magnetic particle concentrator (Life Technologies). The collected glomeruli were used for total RNA isolation.

### Serological and urinary analysis

To evaluate the systemic autoimmune condition, serum levels of anti-dsDNA antibody were measured using the mouse anti-dsDNA Ig (Total A+G+M) ELISA kit (Alpha Diagnostic International, San Antonio, TX, USA). To evaluate renal function, sBUN and sCre levels in all animals were measured using a Fuji Dri-Chem 7000v instrument (Fujifilm, Tokyo, Japan). The uACR was determined using Albuwell M and the Creatinine Companion assay (Exocell, Philadelphia, PA, USA).

### *In situ* hybridization

cRNA probes for *Tlr8* were synthesized in the presence of digoxigenin (DIG)-labelled UTP using a DIG RNA Labelling Kit in accordance with the manufacturer's protocol (Roche Diagnostics, Mannheim, Germany). The primer pairs for each probe are shown in Table 3 (product size: 758 bp). Cryosections (6 μm) were treated with acetylation solution and digested with proteinase K. The sections were incubated with a prehybridization solution and then with a hybridization buffer containing 50% formamide, 10 mM Tris-HCl (pH 7.6), 200 mg/mL RNA, 1× Denhardt's solution (0.02% bovine serum albumin, 0.02% polyvinylpyrrolidone, and 0.02% Ficoll PM400; Sigma-Aldrich, St. Louis, MO, USA), 10% dextran sulphate, 600 mM NaCl, 0.25% SDS, 1 mM EDTA (pH 8.0), and a sense or antisense RNA probe (final concentration, 0.2 μg/mL) for 24 h at 58°C. After washes in saline sodium citrate buffer, the sections were then incubated with 0.2% polyclonal sheep anti-digoxigenin Fab fragments conjugated to alkaline phosphatase (1: 400; Nucleic Acid Detection Kit, Roche Diagnostics) for 24 h at room temperature. The signal was detected by incubating the sections with a colour substrate solution (Roche Diagnostics) containing nitro blue tetrazolium/X-phosphate in a solution composed of 100 mM Tris-HCl (pH 9.5), 100 mM NaCl, and 50 mM MgCl_2_ in a dark room overnight at room temperature.

### Immunofluorescence

In deparaffinized sections, antigen retrieval was performed in 10 mM citrate buffer at 105°C for 20 min. The sections were washed and blocked with 5% normal donkey serum for 60 min at room temperature. The sections were then incubated with rabbit polyclonal antibodies for TLR8 (1:1000; Abcam, Cambridge, UK) and mouse monoclonal antibodies for synaptopodin (1:50; Fitzgerald, Acton, MA, USA) overnight at 4°C. After washes in PBS, the sections were incubated with Alexa Fluor 546-labelled donkey anti-rabbit IgG antibodies (1:500; Life Technologies) and Alexa Fluor 488-labelled donkey anti-mouse IgG antibodies (1:500; Life Technologies) for 30 min at room temperature and then washed again. For nuclear staining, the sections were incubated with Hoechst 33342 (1:2000; Dojindo, Kumamoto, Japan) for 5 min and examined using a fluorescence microscope (BZ-9000; Keyence, Osaka, Japan).

### Reverse transcription and real-time PCR

mRNA expression was analysed as previously described^6^. Briefly, total RNA was isolated from the glomeruli, spleen, and urine using an RNeasy kit (Qiagen, Hilden, Germany). cDNA was synthesized from total RNA by reverse transcription (RT) using the ReverTra Ace reverse transcriptase enzyme (Toyobo, Osaka, Japan) and random dT primers (Promega). cDNA was used in real-time PCR with Brilliant III SYBR Green QPCR master mix and Mx3000P (Agilent Technologies, La Jolla, CA, USA). Gene expression in the glomeruli and spleen was normalized to the expression of actin, beta (*Actb*). The primer pairs are shown in Table 3.

### RT- and TaqMan-based real-time PCR

MicroRNA (miRNA) expression was analysed as described previously^19^. Briefly, total RNA including miRNA in the glomeruli and serum was isolated using an miRNeasy kit (Qiagen). Total RNA was reverse-transcribed using miRNA-specific stem-loop RT primers, reverse transcriptase, RT buffer, dNTPs, and RNase inhibitor according to the manufacturer's instructions (Applied Biosystems, Foster City, CA, USA). Real-time PCR was performed with the resulting cDNA using miR-21-specific TaqMan primers with specific probes (Applied Biosystems), a TaqMan Universal PCR Master Mix (Applied Biosystems), and Mx3000P (Agilent Technologies).

### Statistical analysis

The results were expressed as the mean ± standard error (s.e.) and were statistically analysed using a nonparametric Mann-Whitney *U*-test or Welch's *t*-test (*P* < 0.05). The correlation between two parameters was analysed using Spearman's rank correlation test (*P* < 0.05).

## Author Contributions

J.K. designed and performed experiments and analysed data. O.I., T.H., S.O.-K. and Y.K. designed experiments and analysed data. K.M. and T.N. designed experiments and performed experiments. All authors were involved in writing the paper and had final approval of the manuscript.

## Supplementary Material

Supplementary Information Supplementary material

## Figures and Tables

**Table 1:** Clinical parameters of BXSB and BXSB-*Yaa* mice

		dsDNA (μg/mL)	sBUN (mg/dL)	sCre (mg/dL)	uACR (μg/mg)
BXSB	2 months	148.49 ± 16.07	17.73 ± 0.13	1.56 ± 0.17	60.20 ± 9.04
	4 months	135.43 ± 11.22	29.80 ± 3.50	0.56 ± 0.03	47.35 ± 7.40
BXSB-*Yaa*	2 months	535.15 ± 203.72*	20.20 ± 0.28	2.32 ± 1.03	58.72 ± 9.08
	4 months	890.15 ± 95.44*	38.93 ± 9.92	1.30 ± 0.10	439.93 ± 253.78*

Values are the mean ± s.e.

dsDNA, double-strand DNA antibody; sBUN, serum blood urea nitrogen; sCre, serum creatinine; uACR, urinary albumin-to-creatinine ratio.

*Significantly different from BXSB mice at the same age (Mann-Whitney *U*-test, *P* < 0.05); n ≥ 3.

**Table 2:** Relationship between glomerular *Tlr8* expression and podocyte injury indices

		Glomerular	Glomerular	Glomerular
Value/Parameter	uACR	*Nphs1* expression	*Nphs2* expression	*Synpo* expression
Spearman's rank correlation coefficient	0.847	-0.800	-0.738	-0.801
*P*-value	<0.01	<0.01	<0.01	<0.01

The mRNA expression of podocyte functional markers *Nphs1*, *Nphs2*, and *Synpo* in BXSB/MpJ-*Yaa* mice at 4 months was quantified using real-time PCR. n ≥ 5.

uACR: urinary albumin creatinine ratio.

**Table 3:** Summary of gene-specific primers

Gene	Primer sequence (5′–3′)	Product size	Application
(accession no.)	F: forward, R: reverse	(bp)	
*Tlr1*	F: GTGAATGCAGTTGGTGAAGAAC	125	Real-time PCR
(NM_030682)	R: ATGGCCATAGACATTCCTGAG		
*Tlr2*	F: GAGCATCCGAATTGCATCA	163	Real-time PCR
(NM_011905)	R: CACATGACAGAGACTCCTGAGC		
*Tlr3*	F: GATACAGGGATTGCACCCATA	122	Real-time PCR
(NM_126166)	R: GCATTGGTTTGTGGAAGACAC		
*Tlr4*	F: TTCAGAACTTCAGTGGCTGGA	115	Real-time PCR
(NM_021297)	R: CTGGATAGGGTTTCCTGTCAGT		
*Tlr5*	F: ATGCCAGACACATCTGTGAGA	177	Real-time PCR
(NM_016928)	R: ATCCTGCCGTCTGAAGAACA		
*Tlr6*	F: ATGGTACCGTCAGTGCTGGA	104	Real-time PCR
(NM_011604)	R: TCTGTCTTGGCTCATGTTGC		
*Tlr7*	F: TGACTCTCTTCTCCTCCACCAG	198	Real-time PCR
(NM_133211)	R: TCTGTGCAGTCCACGATCAC		
*Tlr8*	F: GTTATGTTGGCTGCTCTGGTTCAC	203	Real-time PCR
(NM_133212)	R: TCACTCTCTTCAAGGTGGTAGC		
*Tlr8*	F: ATGGAAAACATGCCCCCTCAGTC	758	*in situ* hybridization
(NM_133212)	R: GGACAGTTTCCACTCAGATCTAG		
*Tlr9*	F: GAATCCTCCATCTCCCAACA	181	Real-time PCR
(NM_031178)	R: GGGTACAGACTTCAGGAACAGC		
*Tlr11*	F: CACCATTGTGGAGGGAAGAG	158	Real-time PCR
(NM_205819)	R: TCAGAATGAGGAGAACAGAGCA		
*Tlr12*	F: GAACTTCTGCCTGCTCTGGA	146	Real-time PCR
(NM_205823)	R: AACACGCAGAGTGTGGTACG		
*Tlr13*	F: TCCTCTGTTGCATGATGTCG	182	Real-time PCR
(NM_205820.1)	R: CTGTCTTAGGCATCCAGGTTACA		
*Nphs1*	F: ACCTGTATGACGAGGTGGAGAG	218	Real-time PCR
(NM_019459)	R: TCGTGAAGAGTCTCACACCAG		
*Nphs2*	F: AAGGTTGATCTCCGTCTCCAG	105	Real-time PCR
(NM_130456)	R: TTCCATGCGGTAGTAGCAGAC		
*Synpo*	F: CATCGGACCTTCTTCCTGTG	90	Real-time PCR
(NM_177340.2)	R: TCGGAGTCTGTGGGTGAG		
*Nfkb*	F: GGAGTTTGACGGTCGTGAG	219	Real-time PCR
(NM008689)	R: GGGCCTTCACACACATAGC		
*Il1b*	F: AAGGAGAACCAAGCAACGAC	208	Real-time PCR
(NM008361)	R: AACTCTGCAGACTCAAACTCCAC		
*Il6*	F: TGTATGAACAACGATGATGCAC	137	Real-time PCR
(NM031168)	R: TGGTACTCCAGAAGACCAGAGG		
*Tgfb*	F: AGCCTGGACACACAGTACAGC	125	Real-time PCR
(NM011577)	R: CGACCCACGTAGTAGACGATG		
*Tnfa*	F: CGAGTGACAAGCCTGTAGCC	167	Real-time PCR
(NM013693)	R: GAGAACCTGGGAGTAGACAAGG		
*Ifnb1*	F: CAGCTCCAAGAAAGGACGAAC	138	Real-time PCR
(NM010510.1)	R: GGCAGTGTAACTCTTCTGCAT		
*Ifng*	F: CCTTTGGACCCTCTGACTTG	201	Real-time PCR
(NM008337.3)	R: TTCCACATCTATGCCACTTGAG		
*Actb*	F: TGTTACCAACTGGGACGACA	165	Real-time PCR
(NM007393)	R: GGGGTGTTGAAGGTCTCAAA		

## References

[b1] Ahearn J. M., Liu C. C., Kao A. H. & Manzi S. Biomarkers for systemic lupus erythematosus. Transl Res 159, 326–342 (2012). 22424435 10.1016/j.trsl.2012.01.021

[b2] Lech M. & Anders H. J. The pathogenesis of lupus nephritis. J Am Soc Nephrol 24, 1357–1366 (2013). 23929771 10.1681/ASN.2013010026 PMC3752952

[b3] Santiago-Raber M. L., Laporte C., Reininger L. & Izui S. Genetic basis of murine lupus. Autoimmun Rev 3, 33–39 (2004). 14871647 10.1016/S1568-9972(03)00062-4

[b4] Kimura J. *et al.* BXSB-type genome causes murine autoimmune glomerulonephritis; pathological correlation between telomeric region of chromosome 1 and *Yaa*. Genes Immun 15, 182–189 (2014). 24477164 10.1038/gene.2014.4

[b5] Kimura J. *et al.* Quantitative and qualitative urinary cellular patterns correlate with progression of murine glomerulonephritis. PLoS One 31, e16472 (2011). 21304992 10.1371/journal.pone.0016472 PMC3031591

[b6] Kimura J. *et al.* Close relations between podocyte injuries and membranous proliferative glomerulonephritis in autoimmune murine models. Am J Nephrol 38, 27–38 (2013). 23817053 10.1159/000353093

[b7] Subramanian S. *et al.* A Tlr7 translocation accelerates systemic autoimmunity in murine lupus. Proc Natl Acad Sci U S A 103, 9970–9975 (2006). 16777955 10.1073/pnas.0603912103 PMC1502563

[b8] Kawai T. & Akira S. The role of pattern-recognition receptors in innate immunity: update on Toll-like receptors. Nat Immunol 11, 373–384 (2010). 20404851 10.1038/ni.1863

[b9] Baccala R. *et al.* Sensors of the innate immune system: their mode of action. Nat Rev Rheumatol 5, 448–456 (2009). 19597511 10.1038/nrrheum.2009.136

[b10] Garraud O. & Cognasse F. Platelet Toll-like receptor expression: the link between “danger” ligands and inflammation. Inflamm Allergy Drug Targets 9, 322–333 (2010). 20518724 10.2174/187152810793937991

[b11] Mersmann J. *et al.* Attenuation of myocardial injury by HMGB1 blockade during ischemia/reperfusion is Toll-like receptor 2-dependent. Mediators Inflamm 2013, 174168 (2013). 24371373 10.1155/2013/174168 PMC3859028

[b12] Shichita T. *et al.* Peroxiredoxin family proteins are key initiators of post-ischemic inflammation in the brain. Nat Med 18, 911–917 (2012). 22610280 10.1038/nm.2749

[b13] Eleftheriadis T., Pissas G., Liakopoulos V., Stefanidis I. & Lawson B. R. Toll-like receptors and their role in renal pathologies. Inflamm Allergy Drug Targets 11, 464–477 (2012). 22931389 10.2174/187152812803589994

[b14] Chassin C. *et al.* TLR4 facilitates translocation of bacteria across renal collecting duct cells. J Am Soc Nephrol 19, 2364–2374 (2008). 18753256 10.1681/ASN.2007121273 PMC2588102

[b15] Andersen-Nissen E. *et al.* Cutting edge: Tlr5^-/-^ mice are more susceptible to *Escherichia coli* urinary tract infection. J Immunol 178, 4717–4720 (2007). 17404249 10.4049/jimmunol.178.8.4717

[b16] Zhang D. *et al.* A toll-like receptor that prevents infection by uropathogenic bacteria. Science 303, 1522–1526 (2004). 15001781 10.1126/science.1094351

[b17] Wolfs T. G. *et al.* In vivo expression of Toll-like receptor 2 and 4 by renal epithelial cells: IFN-gamma and TNF-alpha mediated up-regulation during inflammation. J Immunol 168, 1286–1293 (2002). 11801667 10.4049/jimmunol.168.3.1286

[b18] Shigeoka A. A. *et al.* TLR2 is constitutively expressed within the kidney and participates in ischemic renal injury through both MyD88-dependent and -independent pathways. J Immunol 178, 6252–6258 (2007). 17475853 10.4049/jimmunol.178.10.6252

[b19] Ichii O. *et al.* Altered expression of microRNA miR-146a correlates with the development of chronic renal inflammation. Kidney Int 81, 280–292 (2012). 21975861 10.1038/ki.2011.345

[b20] Fabbri M. *et al.* MicroRNAs bind to Toll-like receptors to induce prometastatic inflammatory response. Proc Natl Acad Sci U S A 109, E2110–2116 (2012). 22753494 10.1073/pnas.1209414109 PMC3412003

[b21] Gurkan S. *et al.* Inhibition of type I interferon signalling prevents TLR ligand-mediated proteinuria. J Pathol 231, 248–256 (2013). 24151637 10.1002/path.4235

[b22] Pawar R. D. *et al.* Ligands to nucleic acid-specific toll-like receptors and the onset of lupus nephritis. J Am Soc Nephrol 17, 3365–3373 (2006). 17082246 10.1681/ASN.2006030263

[b23] Fu Y. *et al.* Innate stimuli accentuate end-organ damage by nephrotoxic antibodies via Fc receptor and TLR stimulation and IL-1/TNF-alpha production. J Immunol 176, 632–639 (2006). 16365459 10.4049/jimmunol.176.1.632

[b24] Muzio M. *et al.* Differential expression and regulation of toll-like receptors (TLR) in human leukocytes: selective expression of TLR3 in dendritic cells. J Immunol 164, 5998–6004 (2000). 10820283 10.4049/jimmunol.164.11.5998

[b25] Choubey D. Interferon-inducible Ifi200-family genes as modifiers of lupus susceptibility. Immunol Lett 147, 10–17 (2012). 22841963 10.1016/j.imlet.2012.07.003 PMC3425670

[b26] Osnes L. T., Nakken B., Bodolay E. & Szodoray P. Assessment of intracellular cytokines and regulatory cells in patients with autoimmune diseases and primary immunodeficiencies - novel tool for diagnostics and patient follow-up. Autoimmun Rev 12, 967–971 (2013). 23541481 10.1016/j.autrev.2013.02.003

[b27] Peng G. *et al.* Toll-like receptor 8-mediated reversal of CD4+ regulatory T cell function. Science 309, 1380–1384 (2005). 16123302 10.1126/science.1113401

[b28] Banas M. C. *et al.* TLR4 links podocytes with the innate immune system to mediate glomerular injury. J Am Soc Nephrol 19, 704–713 (2008). 18256364 10.1681/ASN.2007040395 PMC2390962

[b29] Machida H. *et al.* Expression of Toll-like receptor 9 in renal podocytes in childhood-onset active and inactive lupus nephritis. Nephrol Dial Transplant 25, 2530–2537 (2010). 20181802 10.1093/ndt/gfq058

[b30] Bartel D. P. MicroRNAs: target recognition and regulatory functions. Cell 136, 215–233 (2009). 19167326 10.1016/j.cell.2009.01.002 PMC3794896

[b31] Ceribelli A. *et al.* MicroRNAs in systemic rheumatic diseases. Arthritis Res Ther 13, 229 (2011). 21787439 10.1186/ar3377 PMC3239341

[b32] Pan W. *et al.* MicroRNA-21 and microRNA-148a contribute to DNA hypomethylation in lupus CD4+ T cells by directly and indirectly targeting DNA methyltransferase 1. J Immunol 184, 6773–6781 (2010). 20483747 10.4049/jimmunol.0904060

[b33] Iyer S. S. *et al.* Necrotic cells trigger a sterile inflammatory response through the Nlrp3 inflammasome. Proc Natl Acad Sci U S A 106, 20388–20393 (2009). 19918053 10.1073/pnas.0908698106 PMC2787135

[b34] Niemir Z. I. *et al.* Podocytes are the major source of IL-1 alpha and IL-1 beta in human glomerulonephritides. Kidney Int 52, 393–403 (1997). 9263995 10.1038/ki.1997.346

[b35] Takano Y. *et al.* Transcriptional suppression of nephrin in podocytes by macrophages: roles of inflammatory cytokines and involvement of the PI3K/Akt pathway. FEBS Lett 581, 421–426 (2007). 17239861 10.1016/j.febslet.2006.12.051

[b36] Timoshanko J. R., Kitching A. R., Iwakura Y., Holdsworth S. R. & Tipping P. G. Leukocyte-derived interleukin-1beta interacts with renal interleukin-1 receptor I to promote renal tumor necrosis factor and glomerular injury in murine crescentic glomerulonephritis. Am J Pathol 164, 1967–1977 (2004). 15161633 10.1016/s0002-9440(10)63757-1 PMC1615771

[b37] Wickman L. *et al.* Urine podocyte mRNAs, proteinuria, and progression in human glomerular diseases. J Am Soc Nephrol 24, 2081–2095 (2013). 24052633 10.1681/ASN.2013020173 PMC3839551

