# Supplementary material for: Overexpression of Toll-like receptor 8 correlates with the progression of podocyte injury in murine autoimmune glomerulonephritis
Source: Sci Rep. 2014 Dec 3;4:7290. doi: 10.1038/srep07290 (PMC4252901; doi:10.1038/srep07290)
Supplement: Supplementary Information — Supplementary material [file srep07290-s1.pdf]

Overexpression of Toll-like receptor 8 correlates with the progression of podocyte injury in murine autoimmune glomerulonephritis

Junpei Kimura, Osamu Ichii, Kosuke Miyazono, Teppei Nakamura, Taro Horino, Saori Otsuka-Kanazawa, and Yasuhiro Kon

Running Head: TLR8 in autoimmune glomerulonephritis

Affiliations:

[1]Laboratory of Anatomy, Department of Biomedical Sciences, Graduate School of Veterinary Medicine, Hokkaido University, Sapporo, Japan

[2]Laboratory of Molecular Medicine, Department of Veterinary Clinical Sciences, Graduate School of Veterinary Medicine, Hokkaido University, Sapporo, Japan

[3]Section of Biological Safety Research, Chitose Laboratory, Japan Food Research Laboratories, Chitose, Japan

[4]Department of Endocrinology, Metabolism and Nephrology, Kochi Medical School, Kochi University, Nankoku, Japan

*Address correspondence to: Dr Y. Kon, DVM, PhD, Laboratory of Anatomy, Department of Biomedical Sciences, Graduate School of Veterinary Medicine, Hokkaido University, Kita18-Nishi 9, Kita-ku, Sapporo, Hokkaido 060-0818, Japan. Tel.: +81-11-706-5189; Fax: +81-11-706-5189; E-mail: y-kon@vetmed.hokudai.ac.jp

List of Supplementary Information

Supplementary Figure 1.

Supplementary Figure 2.

Supplementary Figure Legends.

Supplementary Table 1.

Supplementary Methods.

Supplementary Figure 1.

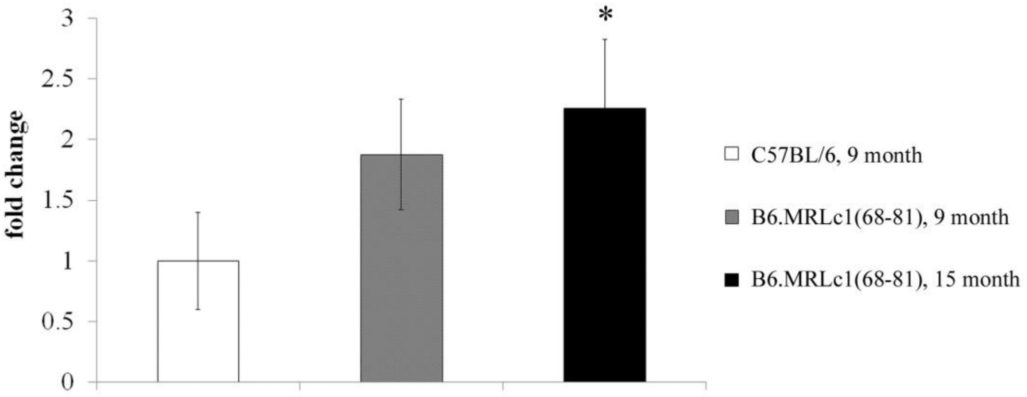

Supplementary Figure 1. Glomerular *Tlr8* expression in B6.MRLc1(68-81) mice. Relative mRNA expression of *Tlr8* in isolated glomeruli from B6.MRLc1(68-81) and control mice. The expression levels were normalized using *Actb*. Values are the mean ± s.e. *, significantly different from control C57BL/6 mice (Mann-Whitney *U*-test, $P < 0.05$); n ≥ 5.

Supplementary Figure 2.

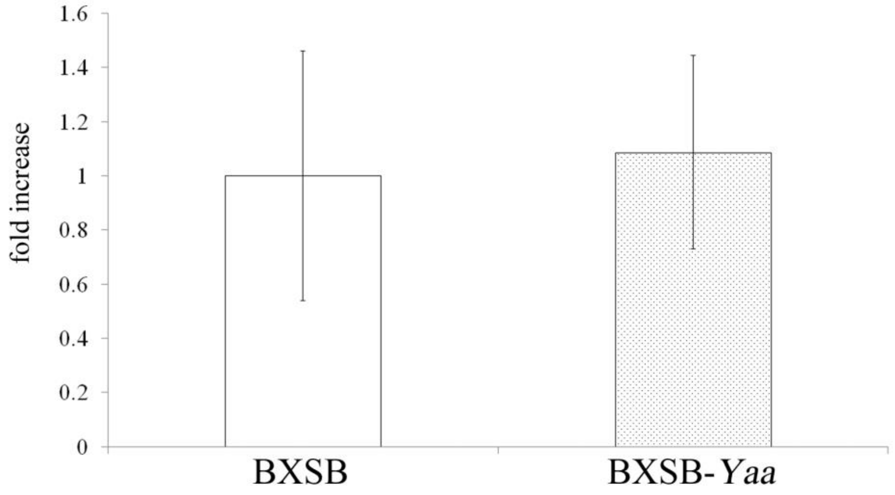

Supplementary Figure 2. Serum *Tlr8* levels in BXSB-*Yaa* mice. Relative *Tlr8* levels in serum from BXSB-*Yaa* mice and BXSB mice at 4 months. Values are the mean ± s.e. n ≥ 4.

Supplementary Table 1. Relationship between glomerular TLR8-mediated cytokine expression and podocyte functional marker expression

Cytokine/podocyte marker	*Il1b*	*Il6*	*Tnfa*
Nphs1	-0.756**	-0.503*	-0.574*
Nphs2	-0.635**	-0.429	-0.515*
Synpo	-0.768**	-0.674**	-0.691**

Values are the Spearman's rank correlation coefficients. * and **, significantly correlated (Spearman's rank-correlation test, *$P < 0.05$. **$P < 0.01$); n ≥ 8.

Supplementary Methods

Glomerular *Tlr8* expression in lupus-prone B6.MRLc1(68-81) mice.

Female B6.MRLc1(68-81) mice at the age of 9–15 months and C57BL/6 mice at the age of 9 months were used as GN model mice and healthy controls, respectively. B6.MRLc1(68-81) mice were created in our laboratory (19), and C57BL/6 mice were purchased from Japan SLC Inc. (Shizuoka, Japan). Briefly, total RNA was isolated from glomeruli using an RNeasy kit (Qiagen, Hilden, Germany). cDNA was synthesized from total RNA by reverse transcription using the ReverTra Ace reverse transcriptase enzyme (Toyobo, Osaka, Japan) and random dT primers (Promega). cDNA was used for real-time PCR with a Brilliant III SYBR Green QPCR master mix and Mx3000P (Agilent Technologies, La Jolla, CA, USA). Gene expression in the glomeruli was normalized to that of actin, beta (*Actb*). The *Tlr8* primer pairs are shown in Table 1.

Serum *Tlr8* levels in BXSB-*Yaa* mice.

Total RNA was isolated from the serum of BXSB mice and BXSB-*Yaa* mice using a miRNeasy kit (Qiagen, Hilden, Germany). cDNA was synthesized from total RNA by reverse transcription using the ReverTra Ace reverse transcriptase enzyme (Toyobo, Osaka, Japan) and random dT primers (Promega). cDNA was used for real-time PCR with a Brilliant III SYBR Green QPCR master mix and Mx3000P (Agilent Technologies, La Jolla, CA, USA). The *Tlr8* primer pairs are shown in Table 1.
